# Knowledge and Attitude of Saudi Physicians Toward Cannabidiol for Pediatric Epilepsy: A Cross-Sectional Study

**DOI:** 10.7759/cureus.36622

**Published:** 2023-03-24

**Authors:** Renad M Alsolamy, Talah Almaddah, Ammar Aljabri, Hadeel A Maaddawi, Faris Alzahrani, Maha Gashlan

**Affiliations:** 1 Medicine, King Saud Bin Abdulaziz University for Health Sciences, Jeddah, SAU; 2 Neurology, King Abdulaziz Medical City, Jeddah, SAU

**Keywords:** knowledge, healthcare professionals, pediatrics, epilepsy, cannabidiol (cbd), cannabis

## Abstract

Background

Epilepsy is one of the most common neurological diseases. Various institutions have shown an interest to investigate the role of cannabidiol (CBD) in treating pediatric epilepsy. CBD is a chemical extracted from the cannabis plant and lacks the euphoria-inducing characteristic. Despite the FDA approval, the physicians' attitude toward CBD is controversial. Therefore, we aim to measure physicians’ knowledge and acceptance of the use of CBD in managing epilepsy patients in Saudi Arabia.

Objectives

The aim of this study is to measure the knowledge and attitude of physicians regarding CBD use in pediatric epilepsy.

Methods

In this cross-sectional study, a validated electronic survey was distributed in the period between September 2021 and October 2021 among pediatricians and neurologists at King Abdulaziz Medical City. The survey consisted of four sections: demographics, the perceived knowledge regarding CBD, the knowledge test, and the attitudes toward CBD. Three-scoring systems were established to assess these sections.

Results

A total of 94 participants were included in this study; 50% of them were males, 81.9% of the participants were in the pediatric field, 13.8% were in the field of neurology, and 4.3% were pediatric neurologists. As for the professional tenure, approximately half of the participants were residents/trainees. Overall, respondents tend to have low perceived knowledge (94.7%) and attitude (93.6%) regarding CBD use. The perceived knowledge and attitude levels were found to be significantly associated with specialty (p < 0.001 and p = 0.001, respectively). Pediatric neurologists had a significantly higher self-assessment score, while pediatricians had the lowest attitude level (p < 0.05). For the knowledge test, surprisingly only one respondent answered all questions correctly, and age was found to be significantly associated with knowledge score (p = 0.001).

Conclusion

This study demonstrates that physicians have poor knowledge and attitude levels regarding the usage of CBD in pediatric epilepsy. Therefore, more education is highly suggested before the introduction of this medication to Saudi patients.

## Introduction

Epilepsy is considered one of the most common neurological diseases, with a global prevalence of approximately 50 million people [1]. Recent studies stated that the estimated figure of epileptic patients in Saudi is 6.54 per 1,000 people [2]. Despite the approval of various anti-epileptic medications over the last two decades, there is still a need to expand the number of medication options for physicians to prescribe since one-third of epileptic patients have drug-resistant epilepsy [3].

Over the last decade, various countries and institutions have shown a huge interest to investigate the role of cannabidiol (CBD) and its efficacy in treating pediatric epilepsy. CBD is a chemical that can be extracted from the cannabis plant and lacks the euphoria-inducing characteristic of tetrahydrocannabinol (THC), another cannabis component. Among these two cannabis constituents, CBD shows superior anti-epileptic potential, which has been proven by multiple studies [4].

The exact mechanism behind CBD’s capability in treating epilepsy is still an area of research; however, several theories have been proposed. One of which is the role of CBD in modulating intracellular calcium through a variety of transient receptor potential (TRP) channels. Furthermore, another study revealed that CBD has an anti-inflammatory action via modulating tumor necrosis factor (TNF) alpha release or inhibiting the reuptake of adenosine, which is also postulated to be the cause behind CBD’s anti-seizure effect [3,4]. The effectiveness of CBD has been supported by various studies. In particular, four randomized control trials and 31 non-randomized studies supported cannabidiol and its potency in decreasing the number of seizures among patients with drug-resistant epilepsy [5,6].

Consequently, the use of CBD in pediatric epilepsy was approved by the US Food and Drug Administration (FDA) in 2018. It is approved to treat seizures in children who fulfill the criteria that state the following: firstly, children who are diagnosed with either Dravet Syndrome (DS) or Lennox-Gastaut Syndrome (LGS), and secondly, children two years of age or older [7]. This medication is considered to be the first FDA-approved drug containing a purified drug substance derived from cannabis. Moreover, CBD is also the first FDA-approved drug for the treatment of patients with Dravet syndrome.

Despite the FDA approval, physicians' attitude toward CBD is controversial. As some physicians support the use of CBD and present it as a treatment option for patients or their families, others are still reluctant to prescribe and suggest this medication. According to a study involving 155 physicians, practitioners only discuss CBD as an option upon request by patients or their parents and only about 40% of them would recommend CBD treatment actively [8].

This division in physicians’ opinions could be attributed to several factors such as the novelty of the supportive evidence. Various studies have been conducted over the last five years to address the knowledge gap regarding this medication; however, there is still a need for more research to unravel the drug characteristics and mechanism of action [5]. Another reason that could be behind practitioners' reluctance in prescribing CBD is the stigma of using it due to the fact of abusing its natural source, cannabis, over the years. According to a recent study that involved 531 dermatologists in the US, 48% of them were concerned about a negative stigma when proposing cannabinoid therapies to patients [9]. Multiple studies have been done worldwide to assess practitioners’ acceptance of prescribing CBD for patients, but none in Saudi Arabia. Therefore, we aim to measure physicians’ acceptance of the use of CBD in managing epilepsy patients in Saudi Arabia.

## Materials and methods

Study area

In this cross-sectional study, an electronic survey hosted through the Google survey webpage was distributed between September 2021 and October 2021 among pediatricians and neurologists along with pediatric neurologists including residents, associate consultants, and consultants at King Abdulaziz Medical City (KAMC) Jeddah, Saudi Arabia.

Data collection method

In light of the available literature, a self-administered questionnaire was constructed with the assistance of a previously published article [10]. The validity of the questionnaire was ensured by performing face validity, content validity, and a pilot study. Also, the reliability of the questionnaire was confirmed through Cronbach's alpha test. The self-assessment section of the questionnaire has yielded reliable results (Cronbach’s alpha = 0.965). Also, the attitude section contained eight items with Cronbach’s alpha = 0.683 making it reliable as well.

Sample size

All those in the field of neurology and pediatrics, regardless of their subspecialty, were invited to contribute to the study. Knowing that the estimated number of physicians at KAMC-J is 95, we calculated the sample size using an online sample calculator (Raosoft) with a 5% margin of error and a 95% confidence level. The required minimum sample size was determined to be 65 subjects. Thus, 94 responses are considered a strongly representative sample.

Measures

The 39-question survey consisted of three sections that included demographics, the physicians’ knowledge regarding cannabidiol (CBD) as a medication, and the respondents’ attitudes toward the usage of CBD. The questions were assessed on a 5-Likert scale with response categories ranging from “Strongly Disagree” (1) to “Strongly agree” (5), combined with a yes/no or true/false response item.

Procedure of analysis

The obtained data from the questionnaire were entered into Microsoft Excel 2016 (Microsoft Corporation, Redmond, WA, USA) and then analyzed using Statistical Package for the Social Sciences, SPSS 23rd version. Frequency and percentages were used to display categorical variables. Minimum, maximum, mean, and standard deviation were used to present continuous variables. Independent t-test and ANOVA test were used to test for the presence of association. ANOVA test was followed by the Tukey post hoc test to determine where the exact difference between groups exists. The level of significance was set at 0.05.

Ethical considerations

This study was conducted after receiving Institutional Review Board (IRB) approval from King Abdullah International Medical Research Center (KAIMRC), Jeddah, Saudi Arabia (IRB approval number: JED-21-427780-119885). The contributions of physicians were voluntary. Before participation in the research, informed consent was gained from all participants.

Scoring systems and assessment of levels

Three-scoring systems were established to assess the participants’ self-assessment of knowledge toward cannabis, actual knowledge toward cannabis, and attitude toward the use of cannabinoids in treating epilepsy in pediatrics. The scoring system of self-assessment of knowledge toward cannabis consisted of 22 items, those who answered not knowledgeable at all/not familiar were given 0 scores, those who answered somewhat knowledgeable/slightly familiar were given 1 score, those who answered moderately knowledgeable/moderately familiar were given 2 scores, those who answered very knowledgeable/very familiar were given 3 scores, and those who answered extremely knowledgeable/extremely familiar were given 4 scores. The total score was then summed, and the total self-assessment of knowledge regarding cannabis was generated for each participant (the lowest possible score was 0, and the highest score was 88). The level of self-assessment of knowledge toward cannabis was then constructed for the participants based on the score, those who had a total score of less than 50% (43 and less) were considered to have a low level, those who had a total score between 50% and 75% (44-66) were considered to have a moderate level, and those who had a total score higher than 75% (67 and more) were considered to have a high level.

The scoring system of the actual knowledge regarding cannabis consisted of four items, those who answered the questions correctly were given a 1 score, and those who answered incorrectly were given a 0 score. The total score was then summed, and the total knowledge score toward cannabis was generated for each participant (the lowest possible score was 0, and the highest score was 4).

The scoring system of attitude toward the use of cannabinoids in treating epilepsy in pediatrics consisted of eight items, those who answered strongly disagree, disagree, or neutral were given 0 scores, those who answered agree were given 1 score, and those who answered strongly agree were given 2 scores. The total score was then summed, and the total attitude toward the use of cannabinoids in treating epilepsy in pediatrics was generated for each participant (the lowest possible score was 0, and the highest score was 16). The level of attitude toward the use of cannabinoids in treating epilepsy in pediatrics was then constructed for the participants based on the score, those who had a total score less than 50% (7 and less) were considered to have a low level, those who had a total score between 50% and 75% (8-12) were considered to have a moderate level, and those who had a total score higher than 75% (13 and more) were considered to have a high level.

## Results

A total of 94 participants were included in this study. Thirteen (13.8%) of them were in the field of neurology, 77 (81.9%) were in pediatrics, and only four (4.3%) of them were in pediatric neurology. As for age, the majority of the participants, 78 (83%), were between 24 and 44 years, 13 (13.8%) were between 45 and 55 years, and three (3.2%) were between 56 and 75 years. Participants were equally divided in regard to gender: 47 (50%) for each gender. As for the participants’ professional tenure, 52 (55.3%) were residents/trainees, five (5.3%) had a tenure of less than five years, 12 (12.8%) had a tenure between six and 10 years, 13 (13.8%) had a tenure between 11 and 20 years, and 12 (12.8%) had a tenure more than 20 years (Table 1).

**Table 1:** Sociodemographic and Academic Profile of the Participants

Characteristics	n	
Age		
24-44 years	78	83.00
45-55 years	13	13.80
56-75 years	3	3.20
Gender		
Male	47	50.00
Female	47	50.00
Professional tenure		
Resident/trainee	52	55.30
Less than 5 years	5	5.30
6-10 years	12	12.80
11-20 years	13	13.80
More than 20 years	12	12.80
Specialty		
Neurologist	13	13.80
Pediatrician	77	81.90
Pediatric neurologist	4	4.30

**Figure 1:**
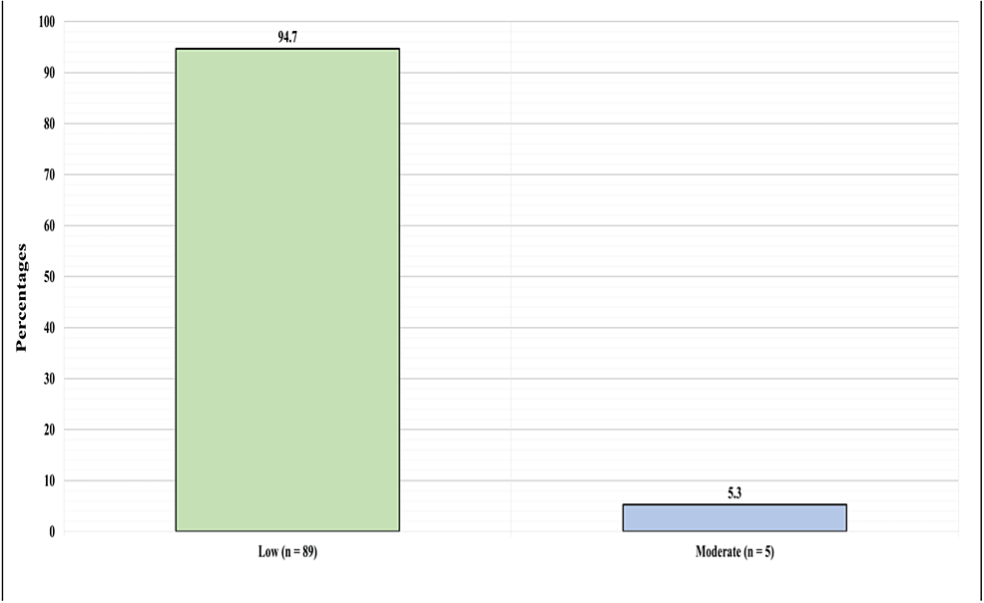
Level of Participants' Self-Assessment of Knowledge Toward Cannabinoid

Overall, physicians demonstrated low levels of perceived knowledge as their mean score was 11.1 + 13.02. Respondents' answers in this section mainly show declined perceived knowledge level. For instance, when asked about the doses of CBD used in clinical trials versus those used in non-FDA-approved CBD products, 72 (76.6%) declared their total lack of knowledge by choosing “Not knowledgeable at all” and 14 (14.9%) were somewhat knowledgeable, while only one (1.1%) physician chose very knowledgeable. Upon applying the scoring system, 89 (94.7%) had a low level of perceived knowledge, while only five (5.3%) had a moderate perceived knowledge of CBD and none had a high level (Figure 1 and Table 2).

**Table 2:** Participants' Self-Assessment About Knowledge Toward Cannabinoid CBD, cannabidiol; THC, tetrahydrocannabinol.

Question	n	%
The effect of the endocannabinoid receptor activation on neuronal function		
Not knowledgeable at all	49	52.1
Somewhat knowledgeable	26	27.7
Moderately knowledgeable	15	16
Very knowledgeable	4	4.3
The proposed mechanisms of action of how cannabinoids affect action within the central nervous system		
Not knowledgeable at all	47	50
Somewhat knowledgeable	28	29.8
Moderately knowledgeable	16	17
Very knowledgeable	2	2.1
Extremely knowledgeable	1	1.1
The differences between endocannabinoids, synthetic cannabinoids, and phytocannabinoids		
Not knowledgeable at all	65	69.1
Somewhat knowledgeable	16	17
Moderately knowledgeable	11	11.7
Very knowledgeable	2	2.1
The mechanism of action of CBD		
Not knowledgeable at all	55	58.5
Somewhat knowledgeable	23	24.5
Moderately knowledgeable	14	14.9
Very knowledgeable	2	2.1
The mechanism of action of tetrahydrocannabinol (THC)		
Not knowledgeable at all	62	66
Somewhat knowledgeable	20	21.3
Moderately knowledgeable	10	10.6
Very knowledgeable	2	2.1
The mechanism of action of phytocannabinoids other than CBD and THC		
Not knowledgeable at all	71	75.5
Somewhat knowledgeable	16	17
Moderately knowledgeable	5	5.3
Very knowledgeable	2	2.1
The bioavailability of cannabis-derived products if administered through inhaled, vaporization, or enteral routes		
Not knowledgeable at all	61	64.9
Somewhat knowledgeable	19	20.2
Moderately knowledgeable	12	12.8
Very knowledgeable	2	2.1
How cannabis-derived products are metabolized		
Not knowledgeable at all	58	61.7
Somewhat knowledgeable	18	19.1
Moderately knowledgeable	15	16
Very knowledgeable	3	3.2
The protein binding capacity of cannabis-derived products		
Not knowledgeable at all	65	69.1
Somewhat knowledgeable	18	19.1
Moderately knowledgeable	11	11.7
The effects of phytocannabinoids on hepatic enzymes		
Not knowledgeable at all	60	63.8
Somewhat knowledgeable	21	22.3
Moderately knowledgeable	11	11.7
Very knowledgeable	2	2.1
Issues with the labeling accuracy of active ingredients in hemp and artisanal cannabis-derived products		
Not knowledgeable at all	71	75.5
Somewhat knowledgeable	14	14.9
Moderately knowledgeable	7	7.4
Very knowledgeable	2	2.1
How hemp and non-FDA-approved (artisanal) cannabis-derived products are produced		
Not knowledgeable at all	71	75.5
Somewhat knowledgeable	15	16
Moderately knowledgeable	6	6.4
Very knowledgeable	1	1.1
Extremely knowledgeable	1	1.1
The half-life of different cannabis-derived products		
Not knowledgeable at all	67	71.3
Somewhat knowledgeable	19	20.2
Moderately knowledgeable	5	5.3
Very knowledgeable	2	2.1
Extremely knowledgeable	1	1.1
The likelihood of cannabis-derived products' transmission through breast milk		
Not knowledgeable at all	53	56.4
Somewhat knowledgeable	26	27.7
Moderately knowledgeable	15	16
The effects of cannabis-based products on the fetus during pregnancy		
Not knowledgeable at all	41	43.6
Somewhat knowledgeable	32	34
Moderately knowledgeable	17	18.1
Very knowledgeable	4	4.3
Overall CBD concentration in FDA-approved versus non-FDA-approved (artisanal) products		
Not knowledgeable at all	74	78.7
Somewhat knowledgeable	11	11.7
Moderately knowledgeable	9	9.6
The concentration of CBD in FDA-approved purified CBD product		
Not knowledgeable at all	73	77.7
Somewhat knowledgeable	12	12.8
Moderately knowledgeable	8	8.5
Very knowledgeable	1	1.1
Potential role of terpenes in enhancing the effect of cannabis-derived products		
Not knowledgeable at all	74	78.7
Somewhat knowledgeable	15	16
Moderately knowledgeable	4	4.3
Very knowledgeable	1	1.1
The uses of FDA-approved purified CBD product		
Not knowledgeable at all	58	61.7
Somewhat knowledgeable	23	24.5
Moderately knowledgeable	9	9.6
Very knowledgeable	3	3.2
Extremely knowledgeable	1	1.1
The doses of CBD used in clinical trials versus those used in non-FDA-approved CBD product		
Not knowledgeable at all	72	76.6
Somewhat knowledgeable	14	14.9
Moderately knowledgeable	7	7.4
Very knowledgeable	1	1.1
The adverse effects of recreational cannabis on a short-term user		
Not familiar	40	42.6
Slightly familiar	29	30.9
Moderately familiar	16	17
Very familiar	7	7.4
Extremely familiar	2	2.1
The dosing of cannabis-based products		
Not familiar	73	77.7
Slightly familiar	13	13.8
Moderately familiar	8	8.5
Self-Assessment Scoring (Maximum possible = 88, lowest possible = 0)		
Mean	11.1	
Standard deviation	13.02	
Minimum	0	
Maximum	52	

As shown in Table 3, a structured knowledge test was part of the questionnaire to measure physicians’ knowledge objectively. The minimum knowledge score was 0, the maximum was 4, and the mean was 1.59 + 0.9. The majority of respondents answered either one or two questions correctly with a percentage of 34 (36.2%) and 36 (38.3%), respectively. Thirteen (13.8%) had three correct answers, and surprisingly, only one (1. 1%) respondent was able to answer all four questions correctly. While 10 (10.9%) physicians were unable to answer any questions correctly.

**Table 3:** Assessment of Participants' Knowledge Toward Cannabinoid CBD, cannabidiol; CBDV, cannabidivarin; THC, tetrahydrocannabinol; THCV, tetrahydrocannabivarin.

Question	n	%
How many different phytocannabinoids are present in the cannabis plant?		
2	7	7.4
Greater than 2, but less than 10	10	10.6
Greater than 10, but less than 50	8	8.5
Greater than 50 (correct)	3	3.2
Don't know	66	70.2
Do you believe the effects of cannabis-based products differ depending on the cannabinoid content (CBD, THC, CBDV, THCV, etc.)?		
Yes (correct)	38	40.4
No	2	2.1
I don't know	54	57.4
Cannabis is legal		
True	10	10.6
False (correct)	74	78.7
I don't know	10	10.6
Isolated plant-derived cannabinoids are legal		
True	19	20.2
False (correct)	34	36.2
I don't know	41	43.6
Knowledge Assessment Scoring (Maximum possible = 4, lowest possible = 0)		
Mean	1.59	
Standard deviation	0.90	
Minimum	0	
Maximum	4	
Number of correct answers		
0	10	10.6
1	34	36.2
2	36	38.3
3	13	13.8
4	1	1.1

In addition, eight questions were asked in an attempt to assess participants' attitudes toward the use of CBD in treating epilepsy. The attitude score was measured as follows: physicians who answered strongly disagree, disagree, or neutral were given 0 scores, those who answered agree were given 1 score, and those who answered strongly agree were given 2 scores. The minimum participants’ attitude score was 0, the maximum was 13, and the mean was 3.18 + 2.59 (Table 4).

**Table 4:** Assessment of Participants' Attitude Toward the Use of Cannabinoid in Treating Epilepsy Affecting Pediatric Patients CBD, cannabidiol.

Question	n	%
1. CBD is effective for epilepsy		
Strongly disagree	1	1.1
Disagree	3	3.2
Neutral	54	57.4
Agree	28	29.8
Strongly agree	8	8.5
2. I favor the use of CBD as a method of treating epilepsy		
Strongly disagree	5	5.3
Disagree	14	14.9
Neutral	61	64.9
Agree	13	13.8
Strongly agree	1	1.1
3. CBD is effective in reducing seizure frequency		
Strongly disagree	2	2.1
Disagree	9	9.6
Neutral	53	56.4
Agree	29	30.9
Strongly agree	1	1.1
4. CBD is effective in reducing seizure severity		
Strongly disagree	3	3.2
Disagree	9	9.6
Neutral	62	66
Agree	18	19.1
Strongly agree	2	2.1
5. CBD is effective in reducing seizure duration		
Strongly disagree	1	1.1
Disagree	8	8.5
Neutral	68	72.3
Agree	15	16
Strongly agree	2	2.1
6. There is a stigma associated with recommending CBD for treating epilepsy		
Strongly disagree	6	6.4
Disagree	2	2.1
Neutral	50	53.2
Agree	25	26.6
Strongly agree	11	11.7
7. I have sufficient knowledge of CBD treatment for epilepsy		
Strongly disagree	26	27.7
Disagree	21	22.3
Neutral	38	40.4
Agree	8	8.5
Strongly agree	1	1.1
8. I need further education about CBD treatment for epilepsy		
Strongly disagree	4	4.3
Neutral	17	18.1
Agree	35	37.2
Strongly agree	38	40.4
Attitude Assessment Scoring (Maximum possible = 16, lowest possible = 0)		
Mean	3.18	
Standard deviation	2.59	
Minimum	0	
Maximum	13	

**Figure 2:**
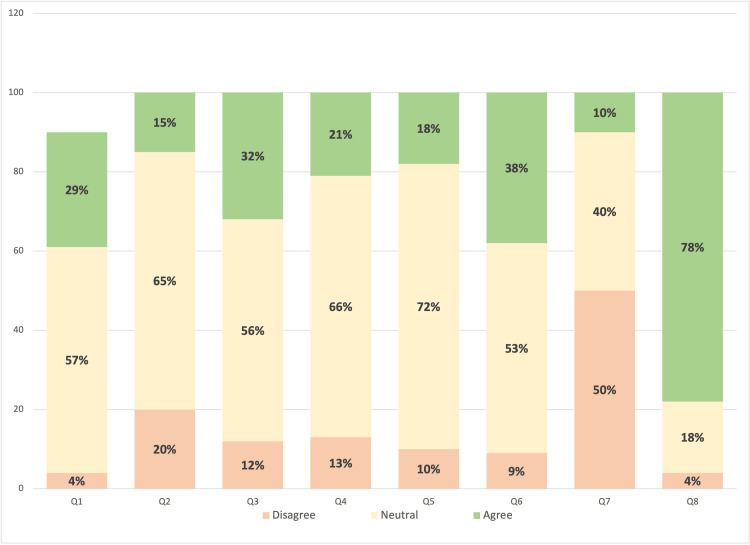
Levels of Participant's Attitude Toward CBD CBD, cannabidiol.

As shown in Figure 2, participants displayed neutral behavior in most of the questions. However, when asked if they feel they need further education about CBD treatment for epilepsy, 73 (77.6%) agreed/strongly agreed with the statement, 17 (18.1%) were neutral, and only four (4.3%) strongly disagreed. Moreover, a discrepancy between physicians’ answers was noted when asked if they think there is a stigma associated with recommending CBD for treating epilepsy. Thirty-six participants (38.3%) agreed/strongly agreed with the statement, approximately half of them, 50 (53.2%), were neutral, and 8 (8.5%) disagreed/strongly disagreed.

Upon measuring physicians' attitude levels, 88 (93.6%) had a low attitude toward the use of CBD in treating epilepsy in pediatrics, five (5.3%) had a moderate attitude, and only one (1.1%) physician was found to have a high attitude. Table 5 shows the factors associated with self-assessment, knowledge, and attitude toward CBD and its use in treating epilepsy in pediatric epilepsy.

**Table 5:** Factors Associated With Self-Assessment, Knowledge, and Attitude Toward Cannabis and Its Use in Treating Epilepsy in Pediatrics *Significant at level 0.05.

Factor	Mean	Standard deviation	p-value
Factors associated with self-assessment about knowledge toward cannabis			
Age			0.205
24-44 years	10.92	12.53	
45-55 years	14.69	16.14	
56-75 years	0	0.00	
Gender			0.747
Male	11.53	13.96	
Female	10.66	12.14	
Professional tenure			0.156
Resident/trainee	8.62	9.49	
Less than 5 years	17.80	17.71	
6-10 years	13.92	15.57	
11-20 years	10.08	11.45	
More than 20 years	17.33	20.34	
Specialty			< 0.001*
Neurologist	18.08	17.18	
Pediatrician	8.35	9.66	
Pediatric neurologist	41.25	9.47	
Factors associated with knowledge assessment toward cannabis			
Age			0.001*
24-44 years	1.71	0.82	
45-55 years	1.23	1.01	
56-75 years	0	0.00	
Gender			0.136
Male	1.72	0.93	
Female	1.45	0.86	
Professional tenure			0.199
Resident/trainee	1.65	0.81	
Less than 5 years	1.60	0.89	
6-10 years	1.75	0.97	
11-20 years	1.69	0.95	
More than 20 years	1.00	1.04	
Specialty			0.346
Neurologist	1.92	0.95	
Pediatrician	1.53	0.90	
Pediatric neurologist	1.50	0.58	
Factors associated with attitude toward the use of cannabinoid in treating epilepsy affecting pediatric patients			
Age			0.122
24-44 years	3.41	2.57	
45-55 years	2.31	2.69	
56-75 years	1	1.00	
Gender			0.906
Male	3.21	2.65	
Female	3.15	2.57	
Professional tenure			0.850
Resident/trainee	2.94	2.22	
Less than 5 years	4.00	3.16	
6-10 years	3.42	2.68	
11-20 years	3.62	2.63	
More than 20 years	3.17	3.86	
Specialty			0.001*
Neurologist	4.69	3.66	
Pediatrician	2.75	2.20	
Pediatric neurologist	6.50	1.92	

As for factors associated with perceived knowledge of CBD, only medical specialty was found to be significantly associated with perceived knowledge (p < 0.001). Tukey’s post hoc test revealed that pediatric neurologists had a significantly higher self-assessment score compared to both pediatricians and neurologists (p < 0.05, in both comparisons). It also revealed that neurologists had a significantly higher self-assessment score compared to pediatricians (p < 0.05). Age, gender, and professional tenure were all not significantly associated with the self-assessment score.

On the other hand, only age was significantly associated with the knowledge test score (p = 0.001) as those aged 56-75 years had a significantly lower knowledge score compared to those who were 24-44 years (p < 0.05). Gender, specialty, and professional tenure were all not significantly associated with knowledge regarding CBD. Furthermore, a significant association between specialty and attitude has been evident (p = 0.001). We found that pediatricians had a significantly lower attitude compared to both neurologists and pediatric neurologists, respectively (p < 0.05 in both comparisons). All other factors were not associated with attitude scores.

Lastly, a Pearson correlation test was performed to measure the association between perceived knowledge level, knowledge test score, and attitude level. As a result, knowledge scores were positively influenced by both perceived knowledge and attitude scores (p = 0.05 and p = 0.002, respectively). Also, a significant association was found between knowledge score and attitude score (p < 0.001).

## Discussion

This study measured the knowledge and attitude of neurologists and pediatrics toward cannabidiol for treating epilepsy among the pediatric age group. Physicians seemed to lack basic knowledge about CBD components, mechanisms of action, and use in clinical practice. Both perceived and actual knowledge scores were considerably low across all domains.

In terms of perceived knowledge, providers scored a mean of 11.1 out of a possible 88. For example, more than half of providers (58.5%) were not familiar with the mechanism of action of CBD, and most (69.1%) did not know the difference between different forms of cannabinoids. This was reflected in the answers on the structured knowledge test where only 14.9% of providers answered more than two questions correctly out of a possible four. These results are in line with previous reports on the significant lack of knowledge regarding cannabis use in clinical practice [11]. Both knowledge scores were strongly associated with age and medical specialty, with those under 44 years and pediatric neurologists achieving higher scores on both tests.

Medical cannabis has proven to be of potential benefit for many conditions, such as epilepsy and amyotrophic lateral sclerosis [12], making it essential to incorporate it into the medical curricula. Many have endorsed cannabis education and recommended providing evidence-based guidelines for medical practitioners as it would promote productive discussions in the healthcare setting [13]. Providers need to be educated on the significant variation among different forms of cannabinoids and their composition. Information provided should also include appropriate preparation, dosing, and route of administration. Moreover, several other factors need to be considered when recommending the use of medical cannabis such as patient variability and condition-specific factors. Cannabis education for medical practitioners is pivotal to providing insight for future research regarding its benefit and ensuring optimal patient care. Knowledge indicators predicted physicians’ attitudes toward CBD use for the treatment of epilepsy.

In our study, attitude toward CBD treatment was significantly influenced by the medical specialty of participants with pediatric neurologists/neurologists scoring higher than pediatricians. However, a large survey-based study by *Epilepsia*, the International League Against Epilepsy (ILAE), reported that the attitudes of general practitioners, nurses, and patients were more favorable than neurologists/epileptologists toward CBD use in epilepsy [14]. All factors included in this study including perceived, actual knowledge, and attitude are strongly influenced by one another and should be addressed together when considering the use of CBD for epilepsy treatment.

For the attitude toward CBD in treating epilepsy, our study showed that 93.6% of our sample displayed a low attitude toward the use of CBD as a treatment option as they scored a mean of 3.18 out of a possible 16. On the other hand, only one physician was found to have a high attitude. Upon answering the section about the efficiency of CBD in several aspects, such as its effectiveness in reducing seizure frequency, duration, and severity, most participants had a neutral perspective on most of the questions. Only 8.5% of the physicians strongly agreed with the statement that “CBD is effective for epilepsy” and less than 15% favor its use for epilepsy treatment. This fact could be an interpretation of lacking knowledge about CBD as 50% of the participants mentioned that they do not have sufficient medical knowledge and, as a result, 77.6% agreed with the statement that they need more education on CBD treatment for epilepsy.

According to the past literature, our study’s results were inconsistent with the other studies. Several studies reported modest to moderate rates toward supporting the practice of medical cannabis ranging from 18% to 60% in their samples. For example, a study conducted by Szaflarski et al. demonstrated that around 60% of neurologists agreed that CBD is effective for epilepsy and about 50% of them consider it as a method of treatment [10]. Nonetheless, other papers showed higher attitude rates (71%-86%) [9,10,13]. Sideris et al. [13] reported that 71% of physicians agreed for cannabis to be an available option to patients. Although the majority of the participants were not registered in the medical marijuana (MMJ) certification program in New York City, they were willing to refer their patients to registered physicians [13]. This discrepancy among different countries and samples could be attributed to several factors, which include legalization issues and the social stigma around cannabis use in the region.

In our study, when physicians were asked about the stigma of prescribing CBD to their patients, 38.3% agreed that stigma exists. The reasons for the stigmatization of cannabis are attributed to several factors such as the public perspective of cannabis as being a recreational drug. As studies showed that public attitudes toward cannabis and its users tend to experience a certain level of stigma in their use of cannabis, especially when dealing with authorities such as landlords, employers, and law enforcement [10,15]. As users continued to experience fear of losing their professional status, being negatively judged on the scale of work performance, or being socially isolated, some countries created programs to authorize the possession of cannabis to face this stigma. For example, the Canadian government officially established Health Canada’s Medical Marihuana Access Regulations (MMAR) in 2001 to authorize the production, supply, and possession of cannabis for therapeutic purposes for individuals who meet specific criteria [15]. Moreover, the stigma could be related to the concerns of illegal activity surrounding cannabis use since the majority of the public lack sufficient knowledge about CBD’s mechanism of action and the fact that it lacks the euphoria-inducing characteristic [3,10]. Studies reported that even participants who had other potentially addictive medications (e.g., oxycontin, sleeping pills) in their management plan were still criticizing the usage of cannabis [15].

Finally, the stigmatization of cannabis could be entangled with other stigmatized vulnerabilities, such as having a history of drug addiction, HIV/AIDS, and mental illness [15]. The previous mechanisms could also be applied when recommending cannabis to a healthcare provider. Studies showed that physicians who consider cannabis as a medicine tend to show lower stigmatization as the ultimate goal is to aid patients by relieving their pain and suffering. On the other hand, physicians who emphasized the lack of scientific evidence and standardization of cannabis as a medicine tend to experience stigma in recommending it, and they tend to adopt the conventional method [16].

As a result, there have been proposals to incorporate education about cannabis in medical schools’ curricula including historical, physiological, legal, and clinical aspects [10,13]. One way to start breaking the stigma is to start legalizing the usage of CBD among providers, as previous studies showed that over 80% of the providers favored the legalization of medical CBD. Moreover, physicians tended to be more comfortable recommending cannabis in states where it is legalized or at health facilities that accepted CBD in the protocol of epilepsy treatment [10]. Further research on the legalization of CBD in Saudi Arabia and its consequences on medical practice and stigmatization among healthcare providers is needed.

Limitations

Our study had several limitations. Our study sample may not be representative, as there were a low number of neurology and pediatric neurology residents; therefore, the findings may not be generalizable to the larger population of healthcare providers. Also, the number of questions in the knowledge section for the distributed survey was limited. This limitation can be a reflection of the fact of evolving knowledge about cannabinoids. Other potential biases include the unreliability of self-administered research and the lack of external validity.

## Conclusions

In conclusion, our findings suggest that providers lack sufficient knowledge regarding CBD use for epilepsy as their scores were low on both knowledge assessments. Moreover, participants demonstrated a neutral attitude toward CBD treatment. We recommend implementing a comprehensive educational program for medical providers before considering CBD treatment for the Saudi population.
